# Taking an insect-inspired approach to bird navigation

**DOI:** 10.3758/s13420-018-0314-5

**Published:** 2018-02-26

**Authors:** David J. Pritchard, Susan D. Healy

**Affiliations:** School of Biology, University of St Andrews, Fife, UK

**Keywords:** Spatial learning, Active vision, Optic flow, Sensory ecology, Landmarks

## Abstract

Navigation is an essential skill for many animals, and understanding how animal use environmental information, particularly visual information, to navigate has a long history in both ethology and psychology. In birds, the dominant approach for investigating navigation at small-scales comes from comparative psychology, which emphasizes the cognitive representations underpinning spatial memory. The majority of this work is based in the laboratory and it is unclear whether this context itself affects the information that birds learn and use when they search for a location. Data from hummingbirds suggests that birds in the wild might use visual information in quite a different manner. To reconcile these differences, here we propose a new approach to avian navigation, inspired by the sensory-driven study of navigation in insects. Using methods devised for studying the navigation of insects, it is possible to quantify the visual information available to navigating birds, and then to determine how this information influences those birds’ navigation decisions. Focusing on four areas that we consider characteristic of the insect navigation perspective, we discuss how this approach has shone light on the information insects use to navigate, and assess the prospects of taking a similar approach with birds. Although birds and insects differ in many ways, there is nothing in the insect-inspired approach of the kind we describe that means these methods need be restricted to insects. On the contrary, adopting such an approach could provide a fresh perspective on the well-studied question of how birds navigate through a variety of environments.

## Introduction

**Fig. 1:**
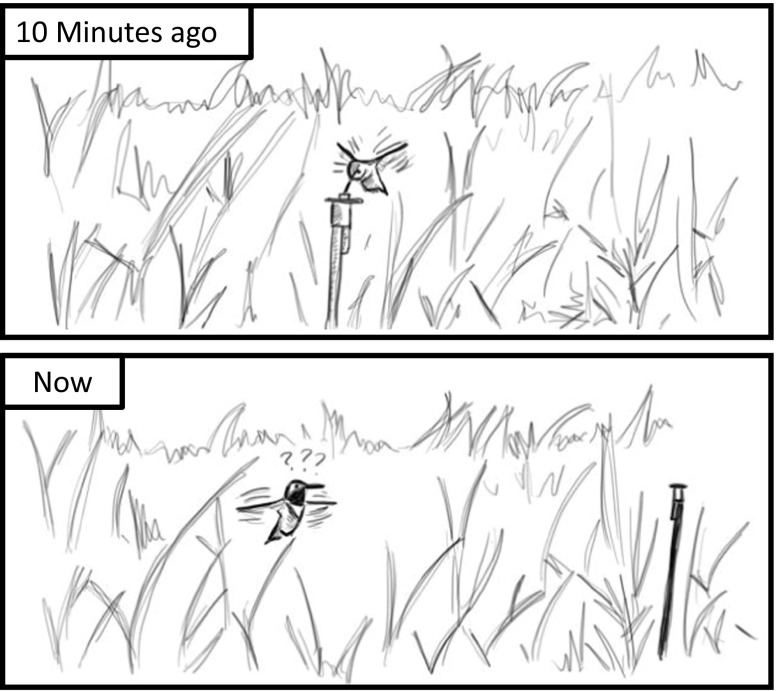
Rufous hummingbirds prioritize spatial information over beacons. When a feeder is moved a short distance, rufous hummingbirds will search where the feeder used to be, even if the feeder is still apparently visible in its new location

It is a chilly May morning in the Westcastle valley, southwestern Alberta, and a male rufous hummingbird sits atop a tall tree. Fifteen minutes earlier he had drunk the sugary contents of an odd-looking flower in the meadow beneath him. Now it is time to feed again. Taking off from his perch, the hummingbird plummets like a stone towards the ground, before pulling up in an elegant arc towards the location of the flower he previously visited. As he approaches close to the flower’s location, however, he stops. The flower is not there. He moves closer, hovering in three-dimensional space, rotating his whole body to scan the scene. Still no flower. After a few seconds, he departs to look for sustenance elsewhere, apparently failing to notice that the flower from which he was expecting to feed is still in the meadow. The flower looks the same. But it has been moved 1 m from its previous position (Fig. 1).

The behavior of this hummingbird is not unusual. A similar phenomenon has been observed many times, not only by researchers interested in the cognitive abilities of these birds, but also by people a little late to put out their hummingbird feeder. Even though no hummingbirds have visited their garden in many months, every year people report seeing hummingbirds hovering and searching around the location at which a feeder had been hung the previous year. The hummingbirds’ apparent prioritization of spatial information over, at least to our eyes, the more obvious color and beacon cues (hummingbird feeders are often red and much larger than the bird), suggests that spatial information is of great importance to them. The question then is: what information is it that the hummingbirds use to remember flower locations?

There has long been a desire to understand how animals remember and navigate to familiar locations (Pritchard & Healy, 2017). Since the very early days in the study of animal cognition and behavior, with the pioneering experiments of Edward Tolman and Niko Tinbergen, biologists and psychologists have studied how animals “know” where they are going. Today, there are several approaches to this question. Inspired by Tolman’s studies on learning and spatial cognition, experimental psychologists, for example, look to uncover the cognitive mechanisms underpinning navigation using well-controlled behavioral experiments to test what animals learn about space. Rather than analyzing behavior in itself, the goal of these experiments is to understand how animals form and use cognitive representations of space, and what information these representations contain. This line of enquiry has focused on how cognitive mechanisms that are seen in other domains, such as attention and associative learning, are used in spatial learning, and how they influence cue use (Cheng & Jeffrey, 2017; Shettleworth, 2009), as well as on investigating the neural basis for this cognition (reviewed in Moser, Rowland, & Moser, 2015), resulting in a Nobel Prize for O’Keefe, Moser, and Moser in 2014. This cognitive approach is one that we ourselves have also taken when investigating foraging in hummingbirds. Taking inspiration from laboratory-based experimental psychologists, we have asked whether hummingbirds use visual “landmarks” to remember a location, what information birds encode about these landmarks, when hummingbirds learn landmarks, and whether these birds rely more on “local” or “global” cues (reviewed in Healy & Hurly, 2013). As a result, we now know that hummingbirds remember which flowers they have visited, and use the spatial location of a flower, rather than its appearance, to remember whether a flower is profitable or not (Hurly & Healy, 1996, 2002; Tello-Ramos, Hurly, & Healy, 2014). Hummingbirds can, however, switch to using the appearance of flowers if spatial cues become unreliable (Flores-Abreu, Hurly, & Healy, 2012; Hornsby, Hurly, Hamilton, Pritchard, & Healy, 2014). Although hummingbirds do not usually rely on a flower’s appearance during navigation, they will use the appearance of the flowers they visit to scaffold the learning of flower locations (Hurly & Healy, 2002) and to generalize information about refill rates of flowers in novel locations (Samuels, Hurly, & Healy, 2014). Hummingbirds also readily use nearby visual landmarks to guide their search. In particular, they appear to use experimental landmarks that occur within 40–50 cm of the flower (Healy & Hurly, 1998; Henderson, Hurly, & Healy, 2006; Pritchard, Hurly, & Healy, 2015; Pritchard, Scott, Healy, & Hurly, 2016), preferring to follow a landmark 5 cm from a flower rather than one 1 m away (Hurly, Fox, Zwueste, & Healy, 2014). They will also use flowers within 40 cm of the goal as landmarks but not flowers that are further than 80 cm away (Healy & Hurly, 1998).

**Fig. 2:**
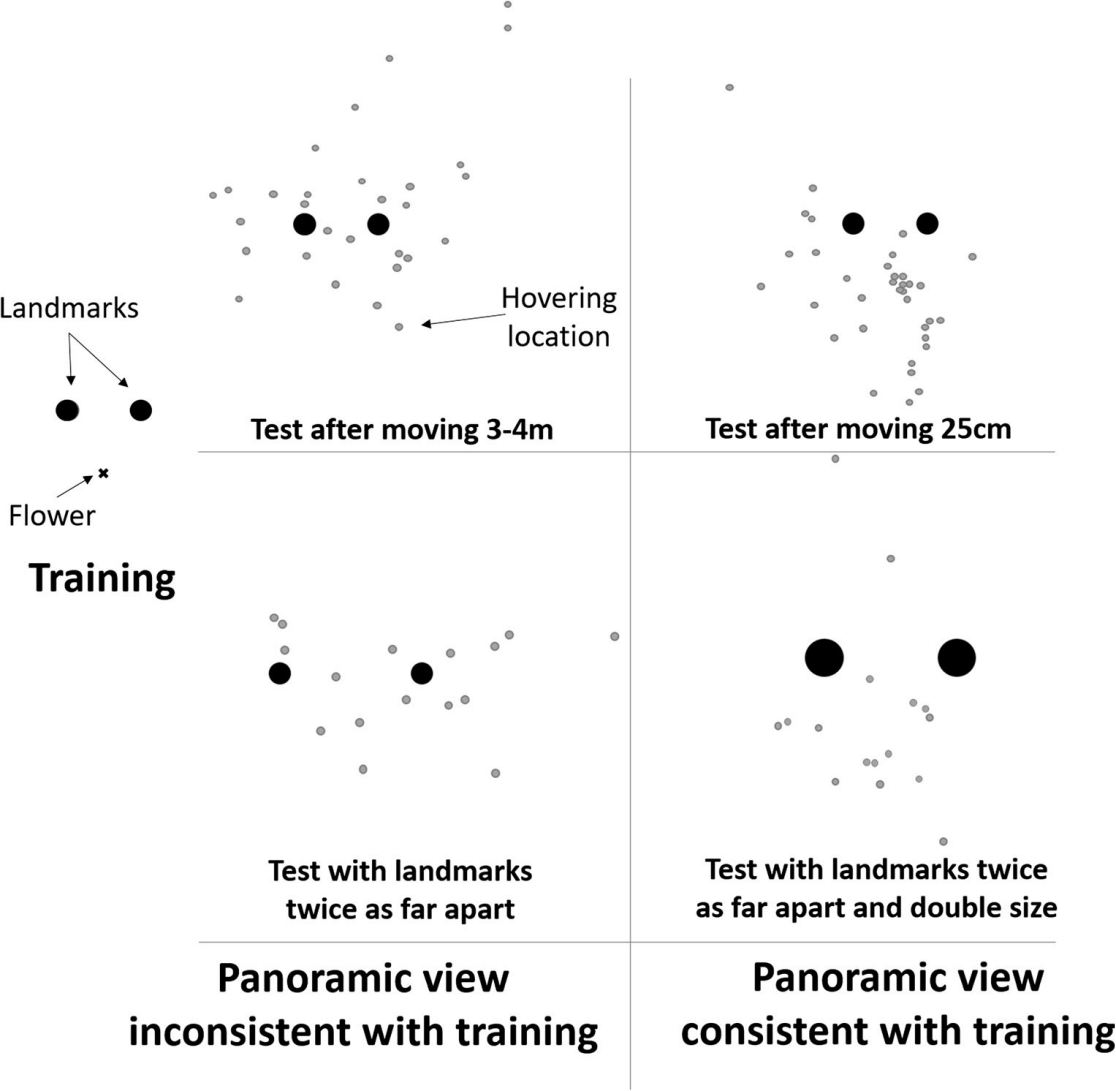
Hummingbirds trained to find a flower is a constant position to a pair of landmarks, will search more on the correct side of the landmarks when the panoramic view is consistent with training, even if the absolute distances between the landmarks have doubled

The use of local landmarks to remember spatial locations is something that wild hummingbirds would appear to share with birds trained and tested in the lab, such as pigeons or nutcrackers (Cheng, Spetch, Kelly, & Bingman, 2006; Gould, Kelly, & Kamil, 2010). There are, however, several key differences in the ways that wild hummingbirds and birds in the lab behave during navigation. This is most apparent in the way that hummingbirds use landmarks. In the laboratory, birds are thought to learn either vectors or directional bearings from one or more landmarks, or to use the relative position of multiple landmarks (Cheng et al., 2006; Gould et al., 2010). When wild hummingbirds have been tested in a similar manner to birds in the laboratory, however, their behavior has not fitted neatly into this framework. In the laboratory, landmarks and goal are moved between trials to ensure that the landmarks, and not other “global” cues, are the best predictor of the goal’s location. Wild hummingbirds, however, searched less accurately as the landmarks and goal were moved further between trials (Pritchard et al., 2015, Fig. 2): the birds searched most accurately when the landmarks were moved only 25 cm between trials, and increasingly less accurately when the landmarks were moved 1 m and 3–4 m between trials. Rather than focusing attention of the landmarks, as training protocols in the laboratory are thought to do, these movements showed that the experimental landmarks provided were not sufficient, in themselves, to guide the hummingbirds’ search. The hummingbirds were similarly disoriented when the landmarks in the array were moved twice as far apart, suggesting that the birds did not learn the position of the feeder from each individual landmark independently (Pritchard et al., in press). The manipulations that resulted in hummingbirds searching in the correct distance and direction were those in which the view of the panorama was most similar to that the birds saw during training, such as when the landmarks and goal were moved only 25cm between trials (Pritchard et al., 2015), or when the size of the landmarks was increased in proportion to the increase in the distance between the landmarks (Fig. 2). In none of these experiments did hummingbirds show any sign of averaging vectors, triangulating compass bearings, or utilize any of the other mechanisms used to explain the behavior of birds in the laboratory. Rather, hummingbirds may relocate their flowers by matching a remembered view, a strategy well-studied in insects, but that has so far been largely dismissed for birds in the lab (Cheng, 1988; Kamil, Balda, & Good, 1999; Kamil & Jones, 1997; Spetch et al., 1997, Lee, Spelke, & Vallortigara, 2012; but see Pecchia et al. 2010, 2011).

The suggestion that hummingbirds use view-based navigation raises two questions. Firstly, how similar is hummingbird navigation to insect navigation, and, secondly, why do wild hummingbirds appear to match views when captive pigeons and nutcrackers predominantly do not. Here we propose that it may be fruitful to take an insect-inspired approach to hummingbird navigation.

## A brief history of insect visual navigation

To understand why insects are thought to match views, and why birds are usually not thought to, we will first take a step back in time. The proposition that insects navigate by matching remembered views first emerged in the 1970s and early 80s. Although the navigational abilities of insects had been studied long before then, by Jean Henri Fabre, Sir John Lubbock, and Georges Romanes in the 19th century, and most notably by Niko Tinbergen and colleagues in the 1930s, the ideas about view-matching really developed when researchers began to consider navigation in terms of how the world looked to navigating insects (Anderson, 1977; Collett & Land, 1975; Wehner & Räber, 1979). Prior to this, it was typically thought that insects learned landmarks or configurations of landmarks, based on experiments similar to those still used to study vertebrate landmark use: animals are trained with a set of landmarks, in tests landmarks are transformed in some way, and the change in the animal’s behavior is used to infer what landmark properties an animal prefers. Just as birds prefer larger, nearer, or more salient landmarks (e.g., Bennett, 1993), *Philanthus* digger wasps also prefer to use large, 3D objects to find their burrow (Tinbergen, 1972). And, as birds (including hummingbirds – Hornsby, Healy, & Hurly, 2017) learn the geometry of an environment, wasps can also use the shape of a landmark configuration, discriminating a circle of landmarks from a square or triangle, for example, to relocate their burrow (Beusekom, 1948).

The move to view-matching navigation in insects began with the discovery that the recognition of a visual pattern depended on the way it mapped onto an insect’s visual field (Wehner, 1972) and was formalized in Cartwright and Collett (1982, 1983)’s influential snapshot model. In this model, Cartwright and Collett suggested that insects navigate by matching their view of surrounding landmarks to an internal template of the angular size and angular bearings of landmarks at the goal. This model not only influenced studies of insect navigation, but also formed the basis for testing view-based navigation in vertebrates. A typical test involved changing the size of a landmark and observing whether the animal searched at a distance that matched the remembered apparent size (Cheng, 1988; Kamil & Jones, 1997; Spetch, Cheng, & MacDonald, 1996a). An animal that continued to search at the same absolute distance from the landmark was considered not to have matched a remembered view.

Although concepts such as the snapshot model were notionally tested in birds and mammals, there was little examination of how these animals behaved during navigation other than to identify the location in which an animal searched. For insects, on the other hand, advances in recording high-speed videos of behavior led to ever more detailed descriptions of how insects moved before reaching the appropriate location. While Tinbergen described wasp homing flights with hand-drawn illustrations (Tinbergen, 1972), 40 years later Collett & Land (1975) analyzed the flights of hoverflies in terms of angles and speed. By quantifying the intricate structures of navigation behavior, researchers could then estimate not only the visual information projecting onto different parts of the eye, but also how this information changed as an animal moved. Understanding how insects moved enabled an *insect-eye view* of navigation, providing new perspective on what spatial information could be contained in a view.

In the past 35 years, static snapshots of distinct landmarks have given way to insects actively interacting with dynamic panoramas encompassing the whole environment. Rather than learning a static snapshot encoding the apparent size and area of specific landmarks, insects also match visual motion cues such as motion parallax (e.g., Dittmar, Stürzl, Baird, Boeddeker, & Egelhaaf, 2010; Lehrer, Srinivasan, Zhang, & Horridge, 1988), using different regions of the eye to assess patterns of optic flow (e.g., Egelhaaf, Boeddeker, Kern, Kurtz, & Lindemann, 2012; Kern, Boeddeker, Dittmar, & Egelhaaf, 2012). Inspired by studies of insects, view-based navigation has also been modelled extensively by insect-researchers and roboticists to test how different types of visual information in views can be used to guide navigation (reviewed in Möller 2012). Models of the visual environment from the perspective of flying and walking insects have suggested that remembered views could take the form of panoramic patterns of light, color, and motion, which would provide both a visual compass along familiar routes (e.g., Baddeley, Graham, Philippides, & Husbands, 2011; Kodzhabashev & Mangan, 2015; Philippides, Baddeley, Cheng, & Graham, 2011; Wystrach, Schwarz, Schultheiss, Beugnon, & Cheng, 2011) and allow insects to pinpoint locations without the need to separate landmarks from the background (e.g., Stürzl, Zeil, Boeddeker, & Hemmi, 2016; Zeil, Hofmann, & Chahl, 2003). This evolution, however, has not been reflected in investigations of visual navigation in vertebrates, particularly in birds, where early tests of the snapshot model in pigeons and nutcrackers seem to have ended almost all discussion of view-matching. The work on landmark navigation in hummingbirds, however, suggests that it would be worth taking inspiration from the work on insect navigation to look again at the ways birds might use views to navigate. Even if hummingbirds do not navigate exactly as bees do, by investigating hummingbirds (and other birds) as we would bees, we might still learn a lot about the use to which birds put visual information to return to a location.

## Modern view of view-matching

The experiments with hummingbirds are not the first to suggest that birds might remember locations by matching learned views. There have been a handful of studies that have also suggested that birds require stable, familiar views of the environment to orient themselves when navigating (Biro, Guilford, & Dawkins, 2003; Pecchia, Gagliardo, & Vallortigara, 2011; Pecchia & Vallortigara, 2010). These data would appear to conflict with those from other experiments in which view-matching in birds in the lab has been tested explicitly. In those experiments, the information that birds used to return to a rewarded location, such as absolute or relative distances, was thought to be incompatible with view-matching (Cheng, 1988; Kamil & Jones, 1997; Spetch et al., 1997). These experiments, however, tested only a single model of view-matching, namely the snapshot model developed by Cartwright and Collett (1983). This model depends on the matching of the apparent size and position of one or two landmarks, and requires an animal to identify landmarks that correspond between the current and remembered views. This focus on matching the apparent size and position of select landmarks could be described as a *landmark-matching* approach to view-matching. Although landmark-matching accurately explains several results seen in bees and ants (e.g., Cartwright & Collett, 1983; Durier, Graham, & Collett, 2003), it is not the only way an animal could use local views to navigate. Over recent decades, other models have emerged (reviewed in Möller 2012, Fig. 3). These alternative models could all still be classed as *view-matching*, in that they describe how animals could return to a location by matching a remembered view, but differ in terms of exactly what information animals are thought to match. Crucially, these models can also explain behaviors that are difficult to explain using landmark-matching alone, behaviors that have previously been argued as evidence against any form of view-matching in birds.

**Fig. 3:**
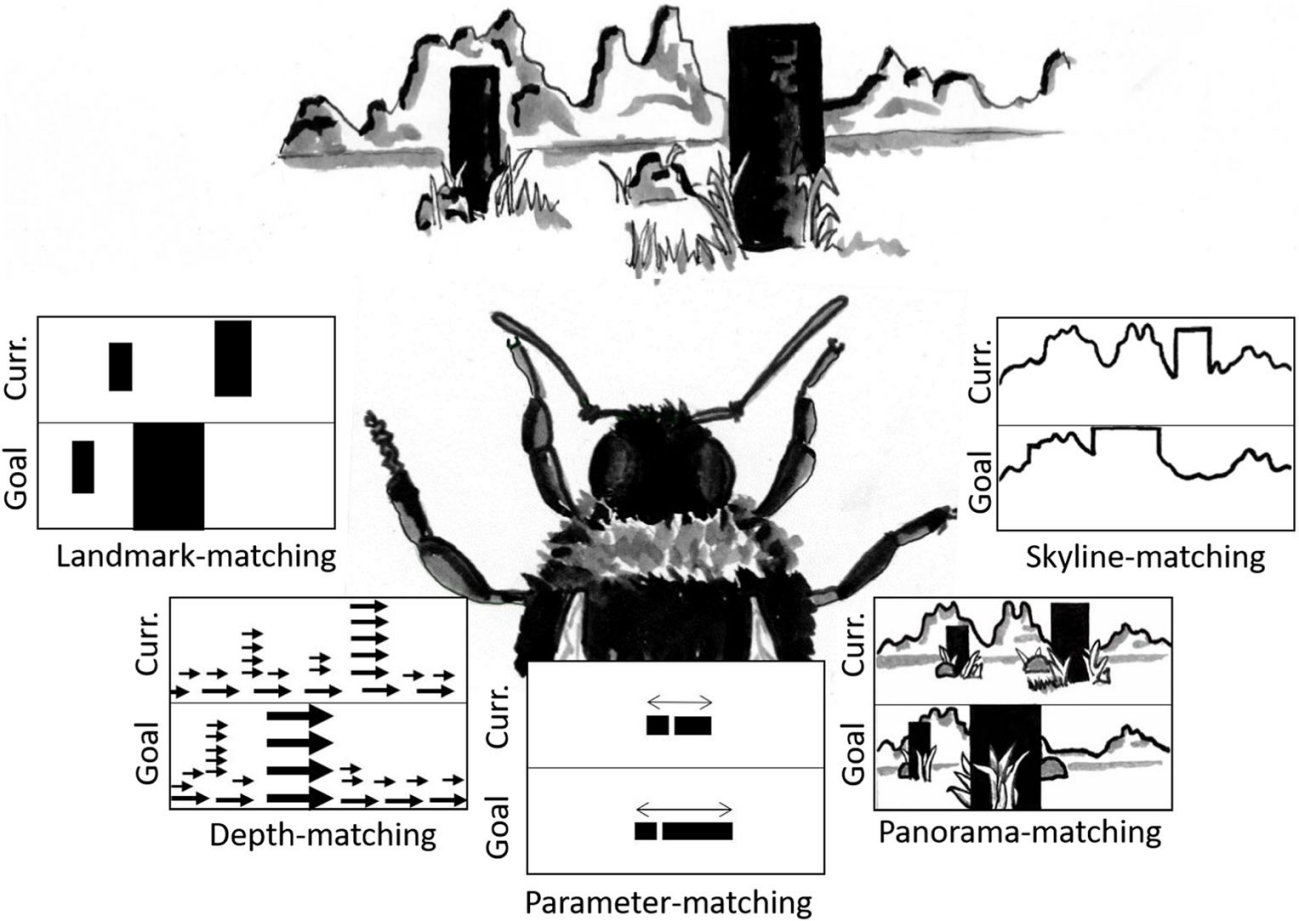
Some of the many suggested ways in which navigating insects could match a remembered view of a goal location

A key prediction of landmark-matching is that animals will search at the location that best preserves either the remembered apparent size or the remembered retinal position of landmarks. When birds searched at the correct absolute distance, this was therefore seen as evidence against view-matching. Even before Cartwright and Collett published their model, however, honeybees had been seen to search at the absolute distance from enlarged landmarks (Cartwright & Collett, 1979), a finding since replicated in wasps (Zeil, 1993b) and other bee species (Brunnert, Kelber, & Zeil, 1994). Rather than using apparent size, these flying insects appear to estimate distance using motion parallax: the relationship between the distance to an object and the speed at which it moves across the visual field, its optic flow (Gibson, 1979; Koenderink, 1986). In addition to sensing the distance of visual objects (Lehrer & Collett, 1994; Lehrer et al., 1988; Srinivasan, Lehrer, Zhang, & Horridge, 1989), bees can also navigate using only patterns of optic flow, searching accurately relative to landmarks that can be seen only when the bees move (Dittmar et al., 2010). This *depth-matching* strategy seems similar to landmark-matching but is based on matching remembered patterns of optic flow rather than learned retinal angles. The “optic-flow snapshot” used by depth-matching insects, could be seen as resembling Marr (1982)’s 2½D sketch for early vision in humans (Cheng, pers. comm.). Indeed, in vertebrates, with access to a wide range of depth cues including stereopsis and accommodation (Harris & Jenkin, 2011; Lazareva, Shimizu, & Wasserman, 2012), a 2½D view of the environment could involve more than just optic flow. Regardless of the exact distance cues contained in a view, by adding depth to remembered views of the environment, depth-matching provides an explanation why insects and other animals might not always use apparent size to estimate distance, and shows that matching local views can still lead to an animal searching at absolute distances from landmarks.

Searching at absolute distances is not the only behavior that is difficult to explain with the snapshot model. In the hummingbird experiments, hummingbirds faced with a panoramic view that resembled that seen during training accurately orient themselves around a pair of landmarks. When the panoramic view differed to that seen in training, however, such as when the landmarks were moved apart but not increased in size, hummingbirds were apparently disoriented. Rather than searching at the location with the closest matching view, such as that preserving the size and position of one landmark or the position of both, hummingbirds searched all around the landmarks (Pritchard et al., in press). The snapshot model would predict that the hummingbirds should have matched either the retinal position of both landmarks, or the apparent size of one of them. However, to explain a similar response by ants to similar landmark manipulations (Wehner & Räber, 1979), Möller (2001) suggested that rather than matching a template, based on apparent size and retinal position, the ants might have matched parameters extracted from the view, such as the *total landmark area*. According to this *parameter-matching* model, when the landmarks were doubled in size, the parameters experienced by the ants along their route remained the same as in training, guiding the ants to the goal. When the landmarks remained the same size, however, ants that attempted to match the parameters learned in training would be drawn closer and closer to one of the landmarks, and would search close to that landmark in an attempt to match the remembered total landmark area. Although Möller’s particular parameter-matching model is now in some doubt (Narendra, Si, Sulikowski, & Cheng, 2007), other derived parameters could still be used in navigation. Lent et al. (2013), for example, demonstrated that ants navigating along a visually-guided route use the remembered *fractional position of mass* in a view, i.e., the proportion of the view to the left and right of their desired heading. In visually complex environments, ants also segmented their view, calculating the centre of mass only from a particular segment rather than the entire scene. Navigation by matching derived visual parameters, such as total landmark area or fractional position of mass, shows that *view-matching* can be much more than just the snapshot model proposed by Cartwright and Collett (1983). And, when it comes to testing visual navigation in birds, that a failure to match the apparent size and retinal position of landmarks is not, in itself, evidence against view-matching.

Both landmark-matching and parameter-matching can involve animals parsing landmarks from the wider panorama. This is not just unique to studies of view-matching; almost all studies of spatial cognition have assumed that birds and other vertebrates learn locations in relation to distinct landmarks. Despite the dominance of landmarks in the literature on animal navigation, visual navigation is also possible without animals identifying landmarks at all (Wystrach et al., 2011). Rather than extracting and recognizing landmarks, an animal could just match the entire visual panorama and move in the direction in which the difference between the current view and the remembered view was the smallest, a form of *panorama-matching.* This is not to say that that animals matching entire panoramas would not be influenced by local landmarks, even if they do not explicitly identify and match them. The shorter the distance a landmark is from an animal, the more the view of the landmark in the panorama will change as the animal moves (Stürzl & Zeil, 2007; Zeil et al., 2003). When matching a view close to the goal, panorama-matching animals would therefore be sensitive to changes in nearby landmarks (e.g., Bennet, 1993, Cheng et al. 1987), not because they have identified and recalled these landmarks, but because these nearby features differ most between the current and remembered views. The idea that the “landmark” is an emergent property of matching whole panoramas has not been considered in most studies of landmark use in birds, but could explain patterns of searching previously ascribed to associative learning favouring some landmarks over others (Wystrach & Graham, 2012).

Over longer distances, ants form habitual routes, using view-matching not to estimate the position of their goal but to keep the ants moving in the correct direction along their route. Ants and bees do this by learning the orientation of the skyline either at the start of their journey or at points along their route (Freas, Whyte, & Cheng, 2017; Graham & Cheng, 2009; Philippides et al., 2011; Towne, Ritrovato, Esposto, & Brown, 2017). In open areas such as deserts or meadows, the skyline changes very little as an animal moves forwards, backwards or side-to-side, but can change dramatically as an animal rotates (Narendra, Gourmaud, & Zeil, 2013; Zeil et al., 2003). As a result, ants and bees can use *skyline-matching* to ensure they are heading in the correct direction, matching the current orientation to that they remember experiencing along the route, with this memory acting as a form of *visual compass*. The skyline can also be used to navigate back from novel locations. Ants knocked off course can return to their route by comparing the height of the skyline to their memory of the skyline on their route (Julle-Daniere et al., 2014; Wystrach, Beugnon, & Cheng, 2012). Once back on their habitual route, the visual compass takes over again and the ant carries on by matching the orientation of the skyline. Skyline-matching, both in terms of the visual compass and skyline height, has been suggested as one reason why some animals, including birds and ants, trained in rectangular boxes confuse rotationally symmetrical corners, even when provided with unique “features” in each corner or along the walls. Regardless of what is in the box, the pattern of the skyline is predominantly determined by the walls and so rotationally symmetrical corners would indicate identical orientations. (Cheung, Stürzl, Zeil, & Cheng, 2008; Stürzl, Cheung, Cheng, & Zeil, 2008, but see Cheng, Huttenlocher, & Newcombe (2013) for the limits of this explanation with some other species).

Rather than a single model with a single defining prediction, i.e. the use of apparent size over absolute distance, view-matching represents a diverse set of strategies. Landmark-matching, depth-matching, parameter-matching, panorama-matching, and skyline-matching, all demonstrate possible ways by which an animal can use local views to return to locations (Fig. 3). These models are not mutually exclusive, and the outcomes from different experiments have supported different mechanisms. This could be because animals use different methods simultaneously, in different environments, or during different stages of navigation. In some cases, this results in animals steering an intermediate course to that predicted by any one method, with the weighting of each mechanism to the overall behavior determined by the variance of each source of information or what an animal has learned (reviewed in Wehner, 2016). This is similar to the compromises seen when navigating birds are faced with conflicting landmark information, and appear to average or integrate information from different sources (e.g., Cheng & Sherry, 1992; Cheng, 1994). Although much of the evidence for visual navigation and landmark use in birds might not fit with predictions based on a strict interpretation of landmark-matching but it is possible that birds could use views in any one (or more) of these other ways to guide their navigation. Testing these models first requires a greater understanding of what visual information is available to navigating birds, and how they might use it.

## Sampling the visual environment

**Fig. 4:**
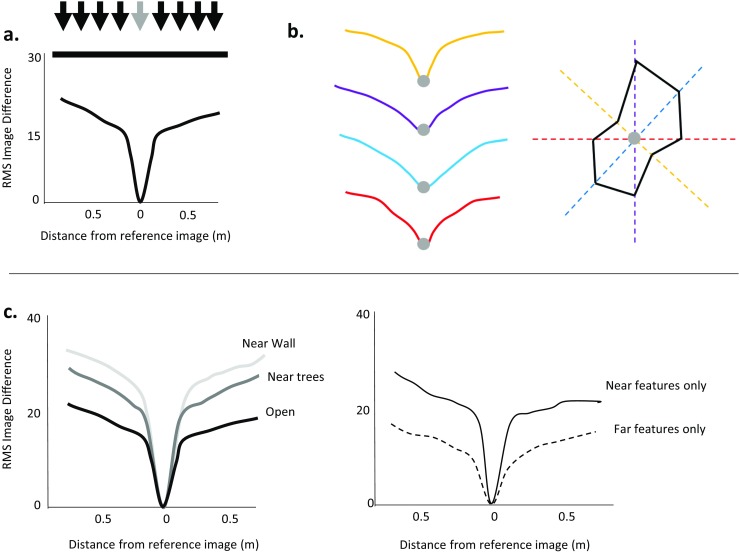
Quantifying the visual environment in terms of image difference functions. (a) The further away a sample image is taken from a goal (black arrows: sample images, grey arrow: goal) the more different the image is from a reference image taken at the goal location. This difference asymptotes after a point, as the distance exceeds the catchment area for that goal location. (b) Image difference functions can be assessed along multiple transects (different color lines), mapping how visual information changes in the area surrounding a goal (inspired by Schultheiss et al., 2013). (c) The shape of the image difference function depends on the structure of the environment (left) as well as what features are compared (right). Nearby features change more with distance from the goal leading to a steeper change in image difference (based on data from Zeil et al., 2003)

Discriminating between different types of view-matching requires understanding where animals might search if they matched different properties within a view. These predictions will depend on what visual information is present in the environment and how this changes along a route or with increasing distance from the goal. Understanding view-matching in animals is therefore linked to understanding their visual ecology, both in terms of what they can perceive, but also importantly, the locations in which they are tested. As a result, many studies of insect navigation over the last 15 years have mapped the visual information available to insects, either by sampling the environment at regular intervals using panoramic cameras (Graham & Cheng, 2009; Mangan & Webb, 2009; Narendra et al., 2013; Philippides et al., 2011) or through constructing 3D computer models of the habitat (Stürzl et al., 2016; Stürzl, Grixa, Mair, Narendra, & Zeil, 2015). This sensory ecology approach fostered the transition from landmark-matching to panorama-matching, which followed Zeil et al. (2003)’s seminal study in which they demonstrated that raw panoramas, without extracted landmarks, at least in theory contained sufficient information for view-matching. When Zeil et al. compared a panoramic image taken at a goal to images taken in the surrounding area, the difference between the goal panorama and the surrounding panoramas increased smoothly with increasing distance from the goal, until it flattens out at the edge of the view’s *catchment area* (Fig. 4a). The slope of this *image difference function* was steeper when a goal was close to prominent landmarks or boundaries, and within an environment the view of nearer features changed more than the view of further features over a comparable distance (Fig. 4c). By capturing the way in which panoramas change with increasing distance in different directions around a goal (Fig. 4b), Zeil et al. demonstrated that it is possible to quantify the visual information present in an environment, and relate changes in behavior to changes in the visual environment. For example, the direction in which an ant heads, and the shape of her search area, is correlated with the shape and slope of visual catchment area in her surrounding environment (Schultheiss, Wystrach, Legge, & Cheng, 2013).

This quantitative approach to evaluating the information available to navigating animals differs from the qualitative approach traditionally taken with birds in the lab. In most studies of birds in the laboratory, researchers have referred to a set taxonomy of different cues, including landmarks and boundaries (Cheng & Sherry, 1992; Kelly, Kamil, & Cheng, 2010; Lee et al. 2012), and local or proximal cues and global or distal cues (Gould-Beierle & Kamil, 1996; Herz, Zanette, & Sherry, 1994; Kelly, Chiandetti, & Vallortigara, 2011; Legge, Spetch, & Batty, 2009). These classes are not mutually exclusive and are based on information that birds could use in a laboratory setting: the walls of the room or edges of the maze provide well-defined boundaries, experimenter-provided objects are local/proximal landmarks, and when animals do not respond to manipulations of these landmarks, they are said to use global or distal cues. Even when researchers painstakingly control exactly what visual information is available to a bird (e.g., Sturz & Katz, 2009), the behavior is still interpreted in terms of using landmarks, global cues, or boundaries. This approach has worked well when testing birds confined to laboratory rooms, but when training and testing hummingbirds in the wild, however, it is far from clear what these terms, developed in the laboratory, might mean. For example, what in the meadow would a foraging hummingbird consider to be a landmark? Although in the past hummingbirds have been trained with artificial “landmarks,” there is no guarantee that the conception of a “landmark” corresponds with the visual information a hummingbird learns or uses. The value, then, of terms such as “local,” “global,” “boundary,” or even “landmark,” terms developed in the laboratory, for investigating navigation by birds or other animals in natural environments is not clear. The sensory ecology-inspired approach, taken by Zeil and colleagues, provides another way of classifying the visual information present in an environment by, for example, using changes in the shape and slope of the catchment area around the goal to analyze the impact of experimental manipulations (e.g., Cheung et al., 2008; Stürzl et al., 2008).

By focusing on how the structure of an environment influences the visual information available to birds, we might also be able to explain why the hummingbirds appear to behave differently to birds tested in the laboratory. Although the scale over which the laboratory birds and the hummingbirds navigate might be quite similar, at least when hummingbirds are searching for a remembered location, the environment is very different. In the mountain valleys in which some of the training and testing of hummingbirds occur, visual cues range from the tufts of grass surrounding a flower, to the mountains several kilometres away. In the laboratory, however, birds are often tested in small walled rooms, with the furthest cues only a few meters away at most. As a result, views will change more rapidly as an animal moves around a laboratory room than they would for an animal covering similar distances in the wild. Quantifying this difference in the visual *information landscape* could be one way to understand the difference between the data acquired from birds trained and tested in the lab and those acquired from hummingbirds trained and tested in the wild.

By examining differences in the slope and shape of the catchment area around the goal, in the style of Zeil and colleagues, and how these are affected by the presence, distance, number, and movement of landmarks, it would be possible to compare the lab and field on an equal footing, rather than make assumptions based on the scale of the behavior. In insect navigation, these sensory ecology methods have led to a *bottom-up* approach to examining spatial cognition rather than the *top-down* approach common in much of comparative cognition (Wystrach & Graham, 2012). This bottom-up approach focuses on understanding how experimental changes affect how the world looks to a moving animal, and how these changes affect that animal’s spatial behavior. Taking such an approach with birds, both in the lab and in the wild, could illustrate how differences in the visual information available to birds affects how birds acquire and use spatial information. To do this, however, we need to look at more than just the environment. We would also need to look closer at the details of behavior.

## Measuring visual consequences of behavior

The information present in a view depends, not only on the environment, but also on how an animal moves through that environment. Some forms of view-matching, such as depth-matching, may even be based on the way an environment appears to move as an insect moves through (Dittmar et al., 2010). Analyzing the details of navigation behavior has a long history in insect navigation, with many studies over the last 40 years taking detailed measurements of spatial behavior. This detail has revealed the important role that behavior plays in determining how insects perceive and learn about space (Egelhaaf et al., 2012). In contrast, most studies of spatial cognition in birds measure only where a bird pecks or digs when searching for a reward, or in a few rare cases, the path a bird took to the goal (e.g., Cheng 1988), or in the case of our hummingbirds, where a bird hovers or which flower he chooses. Behaviors such as hovering, digging, or pecking are used as they are thought to mark a “choice” by the bird, informing us where the bird “thinks” the goal should be (Gould-Beierle & Kamil, 1999; Kamil & Jones, 1997; Kelly, Kamil, & Cheng, 2010; Legge, Madan, Spetch, & Ludvig, 2016; Pritchard et al., 2015; Pritchard et al., 2016; Spetch, Cheng, & MacDonald, 1996). Taking inspiration from insects, however, one might examine not only *where* a bird searches, but *how* they search, including how birds move through the environment and observes their surroundings.

**Fig. 5:**
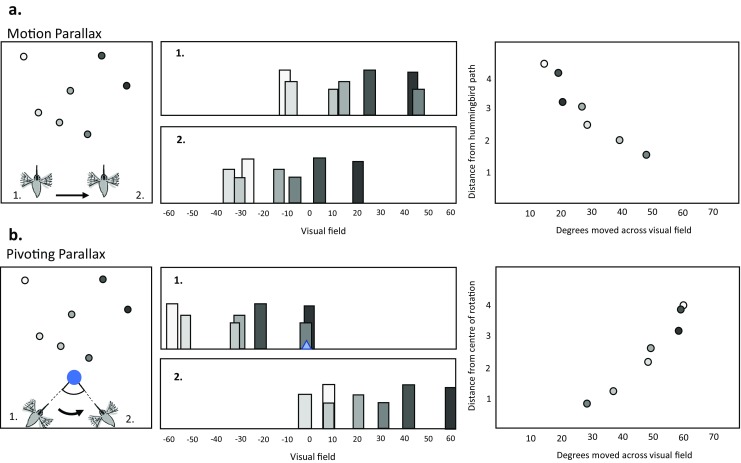
Animals can directly perceive distance by generating parallax. (a) As the hummingbird moves laterally between positions 1 and 2 (left), the relative positions of landmarks in the visual field change due to motion parallax (center). The degree of movement in the visual field is negatively correlated with distance from the hummingbird, closer landmarks appearing to move more than landmarks further away (simulated data, right). (b) When the hummingbird moves between positions 1 and 2, pivoting to keep facing the center of rotation (left, blue circle), the relative positions of the landmarks in the visual field change in a different manner (center, blue triangle: center of rotation). Rather than distance from the hummingbird, the degree of movement across the visual field is positively correlated with distance from the center of rotation, with *further* landmarks moving faster than landmarks closer to the center of rotation (simulated data, right)

Because compound eyes of insects are fixed to the head, insects use head movements to change their view of their surroundings. This interaction between the way in which an insect moves and what she sees means that insects can use specialized movements to directly perceive spatial information such as speed, depth, and proximity, rather than using cognitive resources to compute these properties indirectly. During navigation, insects use a range of different head movements, from the elaborate yet stereotyped fixations and saccades of learning flights, to much more subtle behaviors focused on keeping the head level (Raderschall, Narendra, & Zeil, 2016), generating visual motion (Riabinina, de Ibarra, Philippides, & Collett, 2014; Voss & Zeil, 1998), scanning the environment (Lent, Graham, & Collett, 2010; Wystrach, Philippides, Aurejac, Cheng, & Graham, 2014), or reducing rotational optic flow (Collett & Land, 1975; Hateren & Schilstra, 1999; Schilstra & van Hateren, 1998; Wagner, 1986). These behaviors can be analyzed in detail and interpreted in terms of the ways in which they influence the visual information that insects experience. For example, during orientation flights wasps and bees fly sideways while keeping their gaze facing forwards (Collett, 1995; Stürzl et al., 2016; Zeil, 1993a, 1993b). This movement results in motion parallax across an insect’s field of view, with nearer objects appearing to move faster than objects further away, and so may be involved in mechanism such as depth-matching (Fig. 5a). Other times, wasps and bees fly in an arc around an object or an important location such as a nest, pivoting their gaze direction so that they remain facing the centre of rotation (Voss & Zeil, 1998; Zeil, 1997). Whereas motion parallax results in objects closer to the observing insect appearing to move faster across the view, this pivoting parallax results in objects moving faster the *further* they are, not from the insect, but from the center of rotation (Fig. 5b). Pivoting parallax can therefore provide information about the distance of cues from the nest, or act to visually shear an object from the wider panorama. In these examples, *how* insects search tells us just as much about spatial learning and navigation, as analysing *where* insects search.

**Fig. 6:**
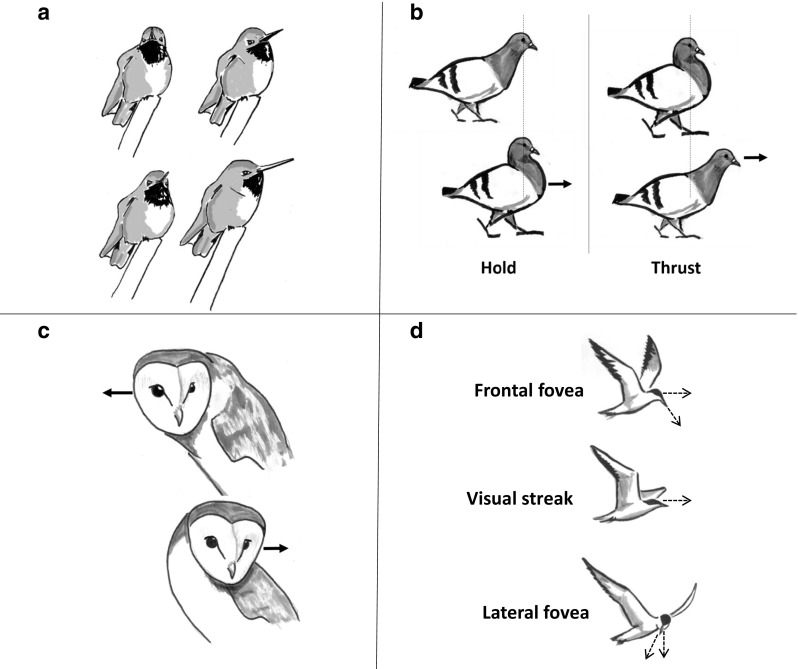
Many birds use head movements to structure the visual information they perceive. (a) hummingbirds and other birds primarily change their gaze direction using fast saccadic head movements. (b) pigeons and other birds bob their heads while moving, holding their heads still as their body moves forward, then thrusting their head in front of their body. (c) owls and other birds make side-to-side peering movements, often when assessing distances. (d) Gull-billed terns (*Gelochelidon nilotica*) flick their head between different positions during hunting, with each position projecting the scene on a different region of the tern’s retina

Although not fixed on their head as insect eyes are, the eyes of many birds, including hummingbirds, are very limited in their movement (Land, 2015), and so instead birds tend to move their heads (Fig. 6a). When unrestrained birds, such as walking chickens, do move their eyes, these movements seem to be in the same direction as larger head movements, acting to complement head movements rather than to look elsewhere (Pratt, 1982). As a result, the way in which a bird moves its head will therefore determine much of the visual information it experiences, just as in insects. Although hummingbirds do not seem to perform anything as stereotyped or obvious as a learning flight, many birds, including hummingbirds, move their heads in particular ways that appear to function as a way to extract spatial information from the visual environment. Pigeons, cranes, and grebes, for example all make characteristic *head-bobbing* movements when walking (reviewed in Necker, 2007) or swimming (Gunji, Fujita, & Higuchi, 2013). These bobs consist of a *hold* phase in which the head remains still in space as the body moves forwards, and a *thrust* phase as the head moves rapidly to a new position in front of the body (Troje & Frost, 2000, Fig. 6b). Just as insects move their heads to stabilize their view of the surrounding or generate optic flow, these bobbing movements both stabilize the image on retina, during the hold phase, and may generate visual motion cues, such as motion parallax, during the thrust phase (Jiménez Ortega, Stoppa, Güntürkün, & Troje, 2009). Birds can also generate motion parallax by *peering*, a behavior in which birds move their head side-to-side while perching (Kral, 2003, Fig. 6c). Because motion parallax provides information about absolute and relative distances, birds could use these movements to perceive and learn spatial information about their environment. Indeed, bobbing and peering movements have been observed in situations in which birds might need to make judgements about space, such as in pigeons landing on a perch (Green, Davies, & Thorpe, 1994) or owls preparing to attack prey (Ohayon, Van Der Willigen, Wagner, Katsman, & Rivlin, 2006).

**Fig. 7:**
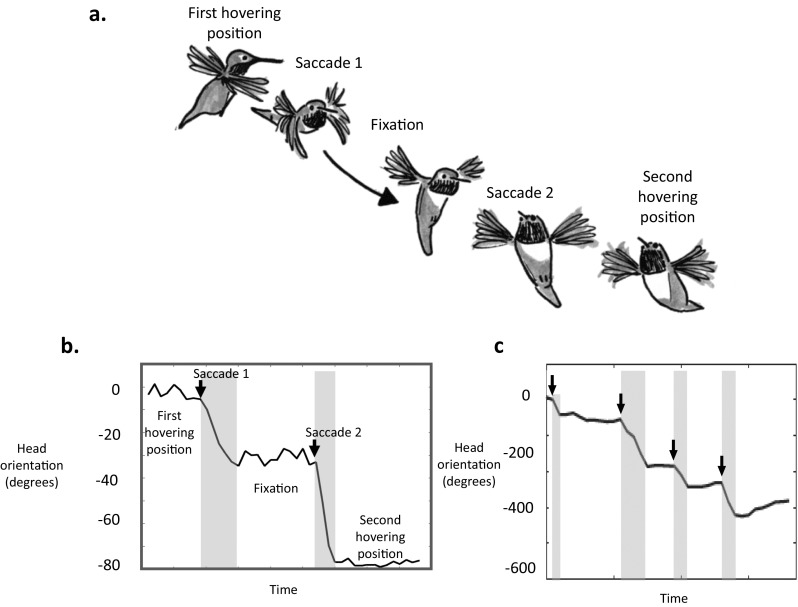
Saccadic head movements in wild hummingbirds. (a-b) hummingbirds moving between hovering positions limit their head rotations to short saccades (arrows on b), keeping their gaze direction fixed between saccades (Pritchard. unpublished data). This pattern of saccades and fixations is similar to that seen in flying insects (c). (Hand-drawn based on data from hoverflies in Zeil, Boeddeker, & Hemmi, 2010)

Flying birds also appear to use behavior to control the patterns of optic flow they experience. Similar to flying insects, flying birds restrict changes in gaze direction to rapid saccades, keeping their head orientation fixed between saccades (Eckmeier et al., 2008; Kress, Van Bokhorst, & Lentink, 2015). When beginning to move away from a previous hovering position, hummingbirds rapidly turn their head to a new orientation (Fig. 7), which they maintain while flying to their next hovering position, rapidly turning their heads once more before stopping and hovering. These saccadic movements could help birds and insects detect depth via motion parallax (Zeil, Boeddeker, & Hemmi, 2008). By limiting head rotations to short bursts, flying birds and insects minimize rotational optic flow, which does not provide information about distances, in favor of translational optic flow, which does provide information about distances. Just as bobbing and peering could allow birds to directly detect distances, restricting head turns to short saccadic bursts could therefore assist flying birds in acquiring spatial information from the environment.

Whereas bobbing, peering, and saccadic turning all involve generating or controlling patterns of optic flow, birds can also use head movements to project features onto different regions of their retina. This is because many birds possess a highly specialized retinal mosaic, with multiple dense regions of photoreceptors, reflecting the visual ecology of the species (Walls, 1942). For seabirds, such as fulmars and manx shearwaters, the world is essentially flat and most important features occur around the horizon. Reflecting this, the largest sensitive region on the retina of a fulmar or shearwater, the area centralis, stretches in a thin band across the visual field, providing highest resolution around the horizon (Lockie, 1952). Other birds, including hummingbirds and pigeons, possess both forward-facing and sideways-facing sensitive regions (Bloch & Martinoya, 1983; Lisney, Wylie, Kolominsky, & Iwaniuk, 2015). These multiple sensitive regions allow birds to see both what is in front of them and in their wider surroundings in high resolution, using these retinal specializations to compensate for their lack of eye movement. Flying terns, for example, use multiple fovea during foraging (Land, 1999) (Fig. 6d). These birds primarily use their temporal fovea to scan the ground, but will occasionally rotate their head 45° to view a recently scanned area with their central fovea. Foraging terns will also flick their heads up, aligning their area centralis with the horizon and use this sensitive band to monitor their surroundings, just as do fulmars and shearwaters. These terns provide a useful case study for how vision in birds is intimately tied to behavior, with head movements enabling birds to view different parts of the environment with different parts of the retina, or examine a single feature using multiple visual regions.

We still know very little, however, about how these different parts of the eye are used during navigation. When studies of avian spatial cognition do consider how birds might use different areas of the visual field (e.g., Spetch et al., 1997), this consideration is rarely in the context of data showing how birds move their heads during navigation. Birds could use this retinal tool kit to simplify the process of recognizing familiar landmarks or locations. Rather than learning or extrapolating all possible view of a landmark, birds could remember how particular sections looked with different parts of the eye when the head is moved in a certain way along a familiar route. This coupling of vision and behavior, is called *active vision*, and has been observed in laboratory chickens trained to recognize an object (Dawkins & Woodington, 2000). During training, chickens made a stereotyped pattern of head movements as they moved along a familiar path, with the details of the pattern and path differing between individuals. Successful recognition of the object across trials involved chickens repeating their sequence of fixating on the object at the same distances, and at the same angles, and with the same eye. When presented with a novel object, chickens approached more slowly, but still showed characteristic large head movements (Stamp Dawkins, 2002). This stereotyped behavior suggests that the chickens recognized the object by recapitulating familiar views along their route, using active vision to simplify the recognition process. Thus far very little is known about how birds might use behavior or active vision to learn about space, mostly because the kinds of quantitative descriptions of head movements that have long been incorporated into work on insect navigation are not used in studies of birds.

## Conclusion

Both birds and flying insects need to navigate 3D space and both rely heavily on visual information to achieve this feat. Insects and birds also both learn about space via associative learning, despite considerable differences in their neuroanatomy. For the last several decades, however, the contents of that spatial memory have been thought to be very different. Data from wild hummingbirds suggest that the difference between insect and hummingbird navigation might not be as large as previously suspected. Whether hummingbirds navigate just like insects is not yet clear. In order to determine whether hummingbirds navigate as insects do, we suggest that it will be fruitful to consider applying the insect-approach to studying spatial cognition outside the lab. By focusing on the information available to navigating animals, an insect-inspired approach to hummingbird navigation could not only help us understand the similarities between hummingbirds and insects, but also provide a way to usefully compare spatial cognition in the lab and the field.

In addition, it is not clear why hummingbirds apear to match views and other birds, tested in the lab under apparently similar conditions, do not. It is clear, however, that modern models of view-based navigation can explain much more of bird spatial behavior than was realized in those early laboratory tests. Recent models of view-based navigation can explain why birds might not use apparent size, why local landmarks can hold such sway over spatial behavior, and why global shape can result in rotational errors. The flexible shift between different forms of skyline-matching in route-following ants also shows how view-based strategies can work together, both in combination or at different stages of navigation. In the absence of either detailed examinations of the head movements of navigating birds, or quantitative sampling of the visual environment in the laboratory, we cannot say for sure that the behaviors currently observed in navigating birds are not the result of some form of view-based navigation.

This could also be true for other vertebrate species. Similar to hummingbirds, wild ground squirrels and chipmunks will search at the previous location of a moved feeder, even when the feeder is clearly visible in its new location. As for traditional studies of birds, this spatial ability has been attributed to cognitive maps based on “remembered metric relations between environmental cues” (Devenport & Devenport, 1994). Using an insect-inspired approach could provide an alternative explanation for such feats of navigation, and just as in birds, there are good reasons to consider such an approach seriously. *Depth-matching* involves learning and matching something akin to the 2½D view of a visual scene described by Marr (1982), in which depth cues such as motion parallax, but also stereopsis, flesh out the image/s acquired by the human retina into an early visual representation of the physical environment. Navigation based on such egocentric 3D views can explain some human landmark use better than explanations based on building 3D representations of the world (Pickup, Fitzgibbon & Glennester, 2013). Models based on view-matching have also been proposed to explain how neural correlates of space, such as head-direction cells, place cells, and grid cells, could deliver robust navigation (Sheynikovich et al., 2009). Although mammals rely more heavily on eye movements than do birds (Land, 2015), mammals still use patterns of head and eye movements to assess distances and depth (Ellard, Goodale, & Timney, 1984; Goodale, Ellard, & Booth, 1990; Wexler & Van Boxtel, 2005), and the technology is now available to record the eye movements of freely navigating mammals (Wallace et al., 2013). The use of sensory ecology methods similar to those used for visual information by Zeil and colleagues (Vanderelst et al. 2016), has also led to the mapping of the acoustic information landscapes available to echolocating bats and to the quantification of the information available for navigation in these environments.

An insect-inspired approach to navigation in birds, and mammals, encourages us to consider vertebrate navigation from a different perspective, and to look more closely at both behavior and the information available to navigating animals. The past 40 years of insect navigation research have provided a toolkit of methods and concepts for testing view-based navigation, recording navigation behavior, and quantifying the information available to navigating animals. Taking inspiration from ideas and methods developed for insects still remains rare for studies of vertebrate cognition (Pritchard, Tello Ramos, Muth & Healy, 2017), but by taking advantage of these tools, and this expertise, we can gain a more holistic understanding of how animals use information to navigate. Not only how a hummingbird in a mountain valley pinpoints a flower’s location with such startling accuracy, but also how birds and other animals learn and navigate within their territory.
